# Modeling and Optimization of Energy Harvesters for Specific Applications Using COMSOL and Equivalent Spring Models

**DOI:** 10.3390/s24237509

**Published:** 2024-11-25

**Authors:** Tharun Reddy Kandukuri, Caizhi Liao, Luigi G. Occhipinti

**Affiliations:** Electrical Engineering Division, Department of Engineering, University of Cambridge, Cambridge CB3 0FA, UK; trk25@cam.ac.uk (T.R.K.); cl2006@cam.ac.uk (C.L.)

**Keywords:** energy harvesting, piezoelectric materials, frequency bands, COMSOL, equivalent spring models, natural frequency, transducer optimization

## Abstract

Energy harvesting from natural sources, including bodily movements, vehicle engine vibrations, and ocean waves, poses challenges due to the broad range of frequency bands involved. Piezoelectric materials are frequently used in energy harvesters, although their effectiveness depends on aligning the device’s natural frequency with the frequency of the target energy source. This study models energy harvesters customized for specific applications by adjusting their natural frequencies to match the required bandwidth. We evaluate commercially available piezoelectric transducers and model them using COMSOL Multiphysics alongside an equivalent spring-mass schematic approach, enabling precise adjustments to optimize energy capture. The proposed system achieves a maximum power output of 160 µW and a power density of 187.35 µW/${\mathrm{cm}}^{3}$ at a natural frequency of 65 Hz. Furthermore, the theoretical maximum power density is calculated as 692.97 W/$m^{3}$, demonstrating the system’s potential for high energy efficiency under optimal conditions. Simulations are validated against experimental data to ensure accuracy. Our findings provide a design framework for optimizing energy harvester performance across diverse energy sources, leading to more efficient and application-specific devices for varied environmental conditions.

## 1. Introduction

Energy harvesting from ambient mechanical sources has emerged as a crucial field of research, primarily due to its potential to energize low-power electronic devices without the need for conventional batteries [1]. Applications of energy harvesting span various domains, including wearables, automotive sensors, and marine monitoring systems, with mechanical energy harvesting from movement, vibrations, or waves representing one of the most interesting options for both the academic and industrial communities [2]. One of the key challenges in optimizing mechanical energy harvesters is ensuring that the harvester’s natural frequency is aligned with the frequency of the target energy source. This alignment is essential for maximizing the energy captured from environmental sources, which often operate within specific and varying frequency bands.

To address this challenge, a growing body of research has focused on optimizing the performance of piezoelectric energy harvesters [3]. Finite element modeling and analysis tools such as COMSOL Multiphysics are commonly employed to design and test energy harvesters. These tools enable researchers to model various parameters—such as the geometry of the harvester, material properties, and environmental conditions—to fine tune the harvester’s natural frequency [4]. Additionally, the use of equivalent spring-mass models provides a simplified way to represent and analyze the dynamic behavior of mechanical systems, helping to predict how a harvester will respond to different mechanical inputs. By simulating these systems before physical prototyping, designers can ensure that the harvester is optimized for its intended application, reducing the time and cost of development.

Different mechanical energy sources, such as human activity, ocean waves, and industrial vibrations, exhibit widely varying frequency ranges. Human motion, for instance, generally generates low-frequency vibrations in the range of 0.5–10 Hz, while ocean waves typically fall within a similar low-frequency range but with higher amplitude due to the larger scale of the movements. Vehicle engines, on the other hand, generate higher-frequency vibrations, ranging from 10 Hz to around 30 Hz, and industrial machinery can produce even higher frequency ranges of up to 200 Hz and beyond. Liang et al. [5] demonstrated how energy harvesters could be tuned to cover these broad frequency bands by using statically balanced, compliant mechanisms that allow for ultra-low, wide-bandwidth vibrational energy harvesting.

**Figure 1:**
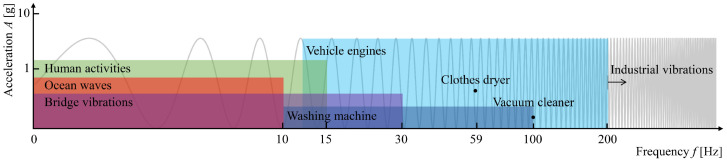
Frequency ranges of different mechanical energy sources. Adapted from Liang et al. [5].

Figure 1 illustrates the frequency ranges of various energy sources that are relevant for energy harvesting:

However, despite the progress in modeling and optimization, many existing methods still rely on complex and expensive solutions to extend the operational bandwidth of piezoelectric harvesters. For instance, Elgamal et al. [6] introduced a multi-frequency, nonlinear broadband piezoelectric energy harvester that operates across a wide range of frequencies, thereby addressing the challenge of frequency mismatch. While effective in expanding the frequency range, the incorporation of nonlinear components significantly increases design and manufacturing costs, making the method less accessible for widespread use. Similarly, Wang et al. [7] proposed a technique using multiple nonlinear adjustments to achieve frequency tuning. Although this approach enhances energy capture across different frequencies, the mechanical complexity required for tuning limits its practicality for users working with off-the-shelf, commercially available transducers.

Another approach, explored by Hashim et al. [8] focuses on optimizing the geometry of piezoelectric cantilevers through COMSOL Multiphysics simulations. Their work demonstrated that altering the shape and dimensions of the cantilever can significantly influence the harvester’s frequency response. However, their study primarily focused on custom-built designs rather than using commercially available piezoelectric transducers. This leaves a gap in practical solutions for users who need to work within the constraints of pre-manufactured components. Additionally, while these approaches address frequency tuning, they often involve significant modifications to the harvester’s structure or the use of complex multi-material systems.

This work addresses the limitations of the existing methods mentioned above by providing a simpler and more cost-effective approach. Instead of relying on nonlinear components or custom-built designs, we focus on shape optimization of the cantilever beam, allowing users to fine tune the natural frequency of commercially available piezoelectric harvesters. This method avoids the need for costly modifications to the transducer itself, offering a more accessible solution for a wide range of applications. Furthermore, by manipulating the geometry of the cantilever beam, we achieve frequency tuning without introducing the complexities seen in previous multi-frequency or nonlinear designs.

Using commercially available piezoelectric transducers is often difficult because they come with predefined natural frequencies that are not easily adaptable to harvest mechanical energy from specifically targeted energy sources [9]. Traditional methods of prototyping energy harvesters require either manipulation of the transducer or the design of a custom-built harvester, both of which present challenges for scalability and cost-effectiveness [10]. Our approach leverages COMSOL Multiphysics simulation capabilities to optimize the design of the cantilever beam that acts as a substrate for the piezoelectric material, enabling frequency tuning without altering the transducer itself [11]. By adjusting parameters such as beam length, width, and thickness, we can model the energy harvester to meet the frequency requirements of the desired energy source, using only materials and components that are readily available.

## 2. Materials and Methods

### 2.1. Materials

The cantilever beam in the piezoelectric harvester system was fabricated using beryllium bronze (alloy c17200, 0.15 mm thick) from Fisher Scientific, Loughborough, UK, chosen for its flexibility and durability under repeated deformation [12]. The DuraAct patch transducer (61 mm × 35 mm × 0.4 mm) from PI (Physik Instrumente) LTD, Cranfield, UK was selected for its efficiency in converting mechanical vibrations to electrical energy [13]. To generate controlled mechanical vibrations, we used an LDS V201 Permanent Magnet Shaker from Hottinger Bruel and Kjaer LTD, Royston, UK [14], driven by a wide-range RS Pro RSDG2082X Arbitrary Waveform Generator from RS London, UK [15]. The signal was amplified using a Cruzer CR-50B power amplifier from Crafter, Gyeonggi-do, Korea.

Electrical data, including the voltage output, were monitored with an Agilent DSO-X 2024A Oscilloscope from Keysight Technologies, Santa Rosa, CA, USA, for high-resolution capture [16]. A laser displacement sensor (OptoNCDT 1220) from Micro-Epsilon, Birkenhead, UK, measured the cantilever’s displacement, essential for determining the natural frequency [17].

Finally, an EmStat4X Potentiostat from Alvatek, Southampton, UK, was employed to conduct linear sweep voltammetry and measure key outputs such as open-circuit voltage and short-circuit current [18], aiding in evaluating the system’s energy harvesting efficiency.

### 2.2. Methods

The experimental setup generated controlled mechanical stimuli for the piezoelectric harvester and analyzed its electrical response. The RS Pro RSDG2082X Arbitrary Waveform Generator produced sinusoidal signals from 25 Hz to 90 Hz, amplified by the CR-50B Power Amplifier to ensure adequate input. This signal drove the LDS V201 Permanent Magnet Shaker, which provided mechanical excitation by vibrating the cantilever beam at set frequencies.

The harvester’s output was routed through a full-bridge rectifier to convert AC to DC, allowing for analysis of power generation capabilities. The Agilent DSO-X 2024A Oscilloscope measured key electrical parameters like open-circuit voltage (OCV) and short-circuit current (SCC), offering real-time performance insights across various frequencies.

A linear sweep voltammetry (LSV) test using the EmStat4X Potentiostat enabled precise power output measurement, characterizing the harvester’s electrical performance across different frequencies and loads to assess energy harvesting efficiency.

To identify the system’s natural frequency, we performed frequency sweeps with the waveform generator while the Laser Displacement Sensor (OptoNCDT 1220) captured the displacement if the cantilever beam’s tip. Using Fast Fourier Transform (FFT) analysis, we pinpointed the peak displacement frequency, which confirmed the resonant frequency necessary for optimizing power output.

Subsequent tests across various input frequencies allowed us to determine the optimal range for maximum power generation. We measured OCV, SCC, and power output, comparing experimental data with COMSOL simulations. Shape optimization tests with magnets on the cantilever examined their impact on stress, displacement, and electrical output. Finally, a 1000 µF capacitor was charged to track the charging curve, providing additional insights into energy storage potential.

## 3. Results

### 3.1. COMSOL Model of Commercially Available DuraAct Piezoelectric Harvester

The commercially available DuraAct piezoelectric harvester, model P-876.A11, was simulated in COMSOL to analyze its stress distribution, natural frequency, and overall mechanical response under mechanical excitation. The properties of the DuraAct transducer, as provided by the datasheet [13], include a minimum lateral contraction of 400 µm/m and a relative lateral contraction of 1.6 µm/m/V. The drive properties indicate an operating voltage range of −50 to 200 V, with PIC255 piezoelectric material type and an electrical capacitance of 150 nF. The blocking force is rated at 90 N, and the minimum bending radius is 12 mm. These parameters were used to create an accurate model of the active piezoelectric layer.

The COMSOL model specifically focused on the active layer of the DuraAct transducer, which is responsible for generating electrical charge when subjected to mechanical deformation. While the simulation provided useful insights into the natural frequency and stress distribution of the active layer, it is important to note that the model does not represent the complete commercial transducer, which includes additional layers such as insulation pads and protective coatings. These additional layers add stiffness and mass to the system, which were not accounted for in the COMSOL model.

**Figure 2:**
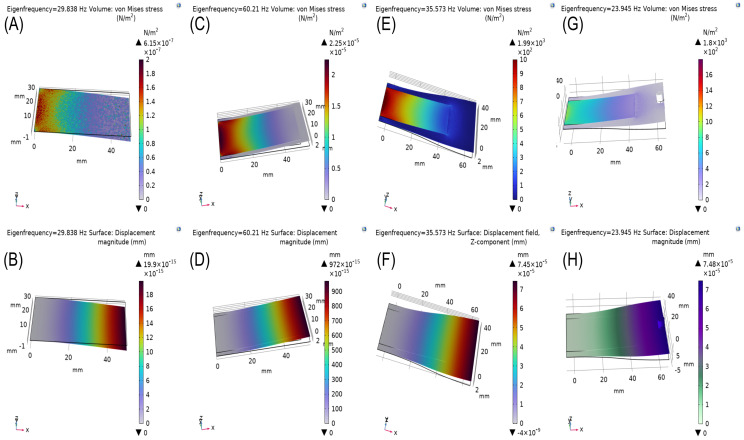
(**A**) Stress distribution for the DuraAct transducer at an eigenfrequency of 29.838 Hz, represented in von Mises stress (N/m^2^). (**B**) Displacement magnitude of the transducer at the same frequency, shown in mm. (**C**) Stress distribution for the DuraAct transducer with a beryllium bronze cantilever beam base at an eigenfrequency of 60.21 Hz, represented in von Mises stress (N/m^2^). (**D**) Displacement magnitude at the same frequency, shown in mm. (**E**) Stress distribution for the DuraAct transducer with a beryllium bronze cantilever beam after shape optimization at an eigenfrequency of 35.573 Hz, represented in von Mises stress (N/m^2^). (**F**) Displacement magnitude of the transducer at the same frequency, shown in mm. (**G**) Stress distribution curve for the optimized cantilever beam with magnets during vibration. (**H**) Natural frequency and displacement curve for the same beam, demonstrating the effect of the added tip mass. All analyses were performed using the COMSOL model, illustrating various configurations and optimizations for energy harvesting.

An offset was observed between the natural frequency in the model and the measured frequency in the real-world prototype. This discrepancy is attributed to the fact that the COMSOL simulation represents only the active layer, whereas the full transducer in the prototype includes additional mechanical components that increase the overall stiffness of the system. Despite this offset, the COMSOL model provides valuable insights into the behavior of the active layer and its role in the energy harvesting process. Figure 2A,B illustrate the baseline stress distribution and displacement magnitude at an eigenfrequency of 29.838 Hz, capturing the response of the transducer prior to any structural modifications. These initial values are critical for understanding the harvester’s inherent characteristics and serve as a reference for subsequent optimization steps.

#### 3.1.1. Simulation Settings

The simulation was conducted using COMSOL Multiphysics 6.2 with a medium mesh size to balance computational efficiency and accuracy in capturing key features like stress distribution and displacement in the cantilever beam. The geometry’s simplicity made finer meshing unnecessary. Multiple materials were used, each with a predefined Young’s modulus and Poisson’s ratio from COMSOL’s material library. The eigenfrequency solver was configured with the ARPACK algorithm, searching around a 1 Hz shift, without geometric nonlinearity. The physics interfaces included solid mechanics, electrostatics, deformed geometry, and shape optimization. Standard COMSOL solver settings were applied with physics-controlled linearization. These configurations ensured computational efficiency while maintaining the fidelity of the results, making the model suitable for real-world applications.

#### 3.1.2. Incorporation of Beryllium Bronze Cantilever Beam

To enhance the mechanical stability and energy harvesting performance of the DuraAct transducer, a beryllium bronze cantilever beam was added as a substrate beneath the piezoelectric layer. Beryllium bronze was chosen for its high strength, durability, and elasticity [12], making it ideal for withstanding repeated deformation under vibrational loads and facilitating efficient energy transfer to the piezoelectric layer, resulting in improved stress and displacement responses.

The substrate was dimensioned to fully cover the DuraAct transducer, with an additional 5 mm margin in both the x and y directions. This extra space allows for placement tolerance during prototype development, ensuring that any minor misalignments do not negatively affect system performance.

Increasing the thickness of a cantilever beam generally raises its stiffness, which, in turn, increases the natural frequency. This can also reduce maximum displacement and strain, factors that are critical for optimal energy harvesting. To minimize this impact, we selected the thinnest commercially available beryllium bronze substrate, allowing us to maintain greater displacement and strain in the piezoelectric layer, thereby enhancing the harvester’s energy conversion efficiency.

The inclusion of the beryllium bronze layer does elevate the system’s natural frequency, which can be advantageous for fine tuning it to the desired operating range. This added flexibility allows the energy harvester to adapt its resonance frequency, making it more suitable for specific vibrational sources in practical applications. The simulation with the thin beryllium bronze substrate demonstrated an increase in stiffness, elevating the eigenfrequency to 60.21 Hz, along with marked improvements in stress and displacement (see Figure 2C,D). This approach aligns with practical, off-the-shelf component availability, enhancing the harvester’s adaptability and performance across a range of applications.

The chosen thickness of the beryllium bronze substrate was carefully selected to balance theoretical predictions with practical testing outcomes, ensuring the design’s resilience and functionality under cyclic loading. Thicker substrates generally improve stiffness and elevate natural frequency, but they may limit the strain and displacement critical for energy conversion. By selecting the thinnest available beryllium bronze substrate, we retained sufficient flexibility to allow the cantilever to experience necessary deformation, optimizing energy transfer to the piezoelectric layer. This choice not only aligns with theoretical expectations for maximizing displacement and strain but also demonstrates robustness in experimental tests, where the substrate endured repeated vibrational loads without compromising energy output or structural integrity. This balance between theoretical design parameters and real-world performance enhances the harvester’s practicality and adaptability, particularly for applications requiring long-term reliability under repetitive mechanical stresses.

#### 3.1.3. Shape Optimization Process

The shape optimization process was driven by the need to create a compact yet efficient energy harvester that could operate within a frequency range of 10–100 Hz. This range includes many common vibrational sources such as vehicle engines and household appliances like washing machines, vacuum cleaners, and clothes dryers. The objective of the optimization was to adjust the dimensions and geometry of the cantilever beam to maximize displacement and energy conversion efficiency while keeping the device within a practical size for real-world applications.

The optimization process is executed until one of the boundary conditions is met. In our case, the most critical condition was the overall compactness of the device. We iteratively adjusted the beam length and geometry to ensure the natural frequency remained within the desired range, but we set a maximum beam length of 65 mm to ensure the final design would remain practical for use in compact applications. By constraining the beam length, we struck a balance between maintaining a low natural frequency and ensuring the device could still be compact and portable.

##### COMSOL Optimization and Boundary Conditions

The COMSOL Multiphysics simulation was integral to our optimization, enabling us to model and refine the cantilever’s geometry by simulating various boundary conditions and material properties. The optimization process was governed by specific boundary conditions, particularly size constraints designed to keep the harvester compact. The device length was constrained to a maximum of 7 cm, with a height limit of 10 cm, ensuring it would be compatible with an existing wearable design. This compact form factor allows the device to be conveniently mounted on a range of energy sources, enhancing its versatility for wearable and portable applications.

#### 3.1.4. Stress and Displacement Analysis After Shape Optimization

The shape optimization of the DuraAct transducer, combined with the beryllium bronze cantilever beam, significantly enhanced the system’s stress distribution and displacement magnitude, leading to improved performance. By reshaping the cantilever beam, the system’s natural frequency was reduced from 60.21 Hz (as shown in Figure 2) to 35.57 Hz, aligning it more closely with the desired range for low-frequency energy harvesting applications. This reduction enables the system to resonate more effectively with lower-frequency mechanical vibrations.

The stress distribution in the newly optimized shape, as shown in Figure 2E, shows an increase by a factor of $10^{8}$, representing a significant improvement over the previous design. This increase in stress enhances deformation in the piezoelectric material, boosting charge generation and energy harvesting efficiency. The optimized shape effectively concentrates maximum stress along the length of the beam, enabling efficient conversion of mechanical vibrations into electrical energy.

Similarly, the displacement magnitude, as shown in Figure 2F, increased dramatically by a factor of $10^{10}$ compared to the pre-optimization design. This increase is critical, as greater displacement correlates with a higher energy conversion potential. The large displacements achieved at lower natural frequencies are essential for maximizing energy output from low-frequency vibrational sources.

In summary, shape optimization successfully enhanced the stress distribution and displacement characteristics, reducing the natural frequency and maximizing energy harvesting potential, as demonstrated in Figure 2.

##### Consideration of Electromechanical Coupling

Electromechanical coupling, a significant factor in piezoelectric energy harvester design, was carefully considered in our optimization process. This coupling, which describes the interaction between the mechanical deformation of the cantilever and the electrical response of the piezoelectric material, directly impacts the resonant frequency and energy conversion efficiency. Various factors contribute to electromechanical coupling effects, including the material properties of the piezoelectric layer, the geometry of the cantilever, and stiffness introduced by connectors and external circuitry. To account for these influences, the COMSOL simulations included material-specific electromechanical properties, ensuring that the model accurately represented how mechanical vibrations would translate into electrical output. By incorporating these coupling parameters, design optimization could be refined to achieve maximum energy harvesting efficiency while ensuring the device remained within the desired frequency range.

To provide a more comprehensive analysis of electromechanical coupling, several additional factors were incorporated into our COMSOL simulations to refine the frequency response and improve energy efficiency. Beyond wire stiffness, which can significantly affect mechanical coupling, the simulation also accounted for boundary conditions, damping effects, and mass loading. Boundary conditions such as fixed or free edges influence the cantilever’s displacement and can shift the natural frequency based on the surrounding structure. Damping effects, including internal material damping, were modeled to understand how energy losses might impact the resonant frequency and energy output over time. Mass loading due to any additional components attached to the system (e.g., magnets or other weights) was also included, as it can alter the effective mass of the cantilever, thereby shifting the resonant frequency. By systematically analyzing these coupling factors, we could further optimize the design to enhance frequency accuracy and energy harvesting efficiency, ensuring that the system would perform reliably under diverse operational conditions.

#### 3.1.5. Impact of Tip Mass on Frequency Tuning

A tip mass was added to the cantilever beam in the form of magnets (5 mm diameter and height) to further reduce the natural frequency while maintaining high stress and displacement levels. Figure 2G shows the stress distribution concentrated near the magnets, amplifying the mechanical deformation. Figure 2H illustrates the natural frequency and displacement curve, with the added tip mass effectively lowering the system’s frequency to approximately 35 Hz, aligning with typical low-frequency vibration sources. This adjustment enables the harvester to resonate more effectively with lower-frequency mechanical inputs, further increasing energy harvesting efficiency.

Collectively, Figure 2A through Figure 2H illustrate the progression of design modifications—from the baseline model to the final optimized configuration with a tip mass—demonstrating how stress distribution, displacement, and frequency tuning improved the energy harvesting potential of the system.

##### Optimization Outcome

**Figure 3:**
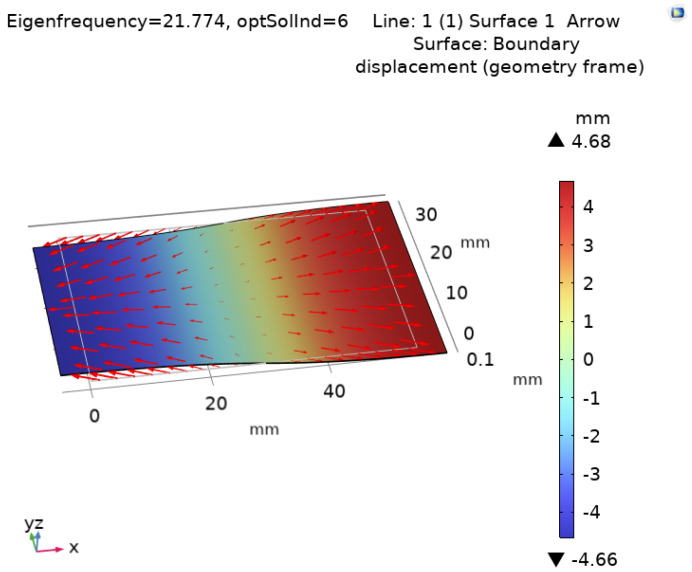
Optimized cantilever beam shape with magnets, showing the direction of displacement (red arrows) and magnitude of change (color gradient).

The final design of the cantilever beam after the shape optimization process is shown in Figure 3. This optimized shape represents the transformation of the cantilever beam that best meets our boundary conditions, balancing size and frequency performance for real-world applications. The addition of magnets provides targeted frequency adjustment by increasing tip mass, lowering the natural frequency to a suitable range for low-frequency vibrations. The final compact design supports easy integration with existing wearable devices and can be mounted on various energy sources, highlighting its adaptability.

It is important to note that while we aimed for a frequency range of 10–100 Hz, we were also aware that adding the electrical circuit in the final prototype could introduce additional stiffness to the system. This increase in stiffness could potentially shift the natural frequency higher than anticipated. Therefore, after multiple iterations, we converged on a solution that limited the beam length to 65 mm, which would allow us to remain within our target frequency range, even after accounting for the additional stiffness introduced by the electrical components.

Had we aimed for a lower frequency, we could have increased the beam length, though this would have resulted in a larger device.

### 3.2. Prototype Fabrication and AC-to-DC Conversion

**Figure 4:**
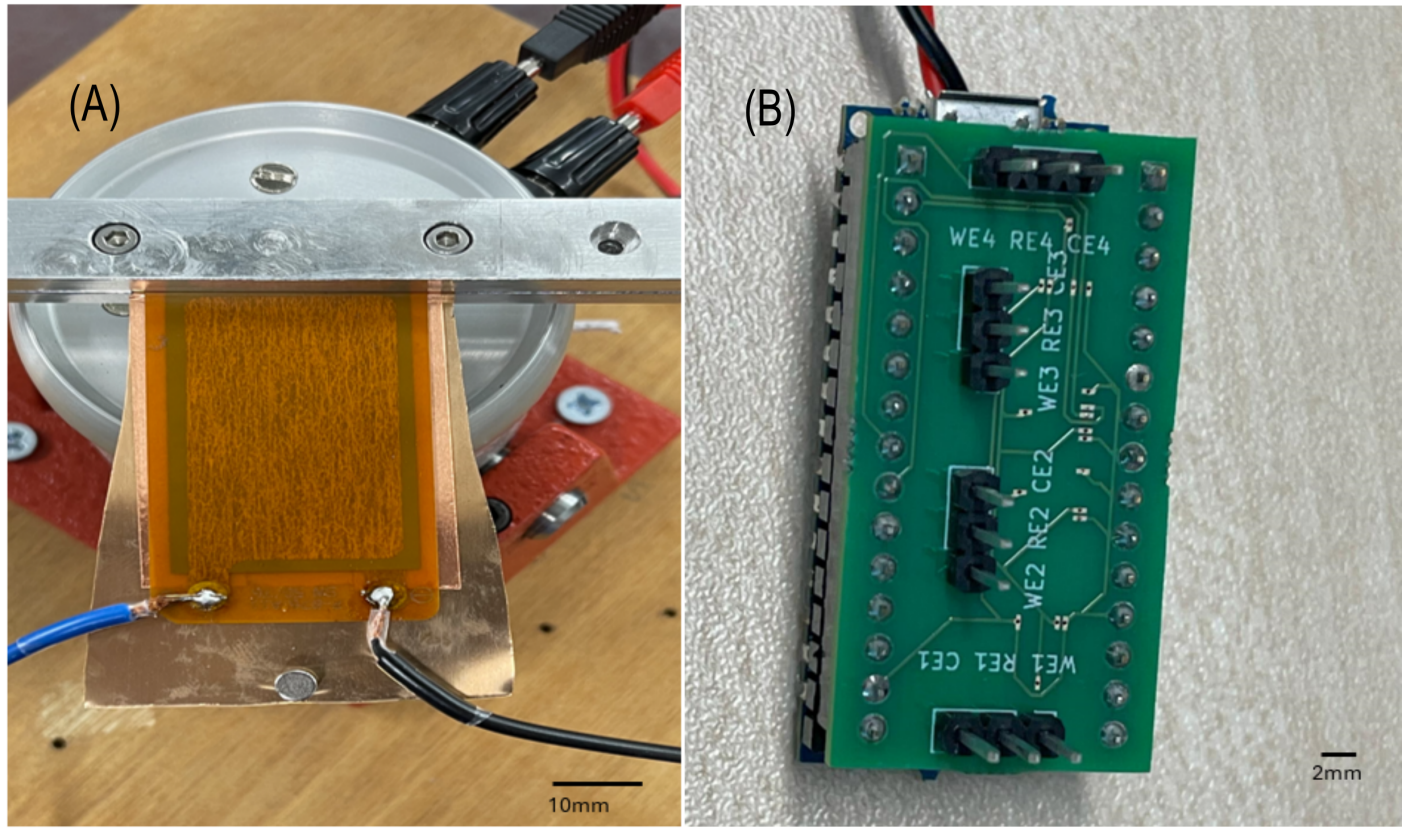
(**A**) Photographs of the fabricated prototype, providing an eagle-eye view and a zoomed-in look. Both images include reference scale bars in the bottom-right corner for size comparison. (**B**) PCB mounted on top of the Arduino Nano, providing Wi-Fi, Bluetooth, and data transfer capabilities. The close-up shows the PCB after mounting, connected to a power source. All images include a reference scale bar in the bottom-right corner for size comparison.

Using the specifications derived from the COMSOL model, we fabricated a physical prototype of the piezoelectric harvester. The DuraAct transducer was mounted on a custom-designed beryllium bronze cantilever beam, matching the dimensions and materials used in simulations. The natural frequency of the prototype was measured with a laser displacement sensor, and an FFT analysis was performed to identify the peak frequency. Figure 4 shows the fabricated prototype setup, which was tested under controlled vibrational conditions.

**Figure 5:**
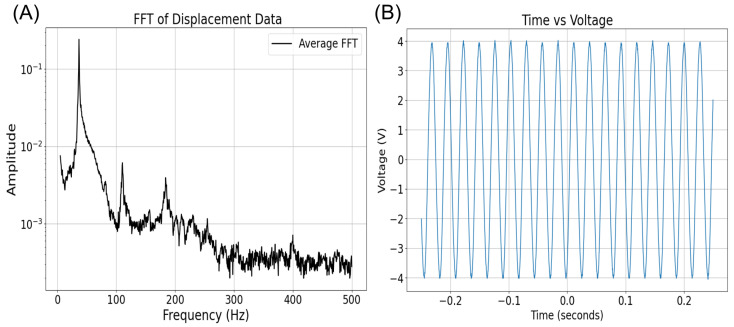
(**A**) FFT curve of the displacement sensor data from the prototype, showing a peak around 35 Hz. (**B**) Voltage output curve of the piezoelectric harvester during vibrational excitation, with a peak–peak open-circuit voltage of 8V.

An AC-to-DC conversion circuit was implemented using a full-bridge rectifier to convert the AC output of the piezoelectric harvester into usable DC voltage. This rectified DC voltage was analyzed to confirm energy harvesting effectiveness in practical applications. Figure 5B presents the voltage output curve of the prototype under resonant excitation, showing a peak output of approximately 3.1 V. This observed voltage aligns closely with COMSOL simulation predictions, validating the prototype’s efficiency.

The fast Fourier transform (FFT) curve generated from the displacement sensor data is shown in Figure 5A, with a peak frequency observed around 35 Hz, slightly deviating from the COMSOL-predicted frequency of 29.8 Hz. This discrepancy is attributed to the additional layers in the commercial transducer, like insulation and protective coatings, which increase stiffness and shift the resonant frequency slightly.

Despite minor variations in frequency and stress due to real-world components, the fabricated prototype’s performance closely matches the COMSOL model’s predictions.

### 3.3. Power Output Testing Under Stimulation with Varying Frequency Vibrations

The prototype was tested for maximum power output under varying vibration frequencies using an RS Pro RSDG2082X function generator and an LDS V201 shaker to provide frequency-controlled mechanical stimulation. A range of frequencies from 25 Hz to 90 Hz was applied to the system, and the power output was measured at each frequency.

**Figure 6:**
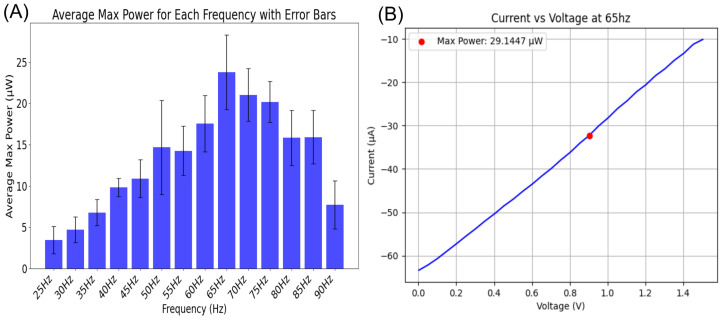
(**A**) Average maximum power vs. frequency plot with error bars, indicating that the system produces peak power at 65 Hz. (**B**) IV curve at 65 Hz showing the current vs. voltage characteristics, with the red dot highlighting the maximum power of 29.1 µW.

The plot in Figure 6A shows the average maximum power output as a function of the vibration frequency, including error bars to represent the variability in power measurements. As observed, the system achieved the highest power output at 65 Hz, with a peak power of approximately 29.1 µW. This frequency likely corresponds to the system’s optimal operating point, though the electrical setup used to measure the power likely introduced some stiffness into the system, which shifted the natural frequency slightly upwards. This will is further explained in the Discussion using a stiffness-mass equivalent of the system.

To further analyze the system at the optimal frequency of 65 Hz, an IV curve was generated, as shown in Figure 6B. The red dot in the IV curve highlights the point of maximum power, calculated as 29.1 µW. This figure provides insight into the current–voltage characteristics at the peak frequency, allowing us to evaluate the electrical performance of the system at its most efficient point.

### 3.4. Capacitor Charging Test for Power Storage

**Figure 7:**
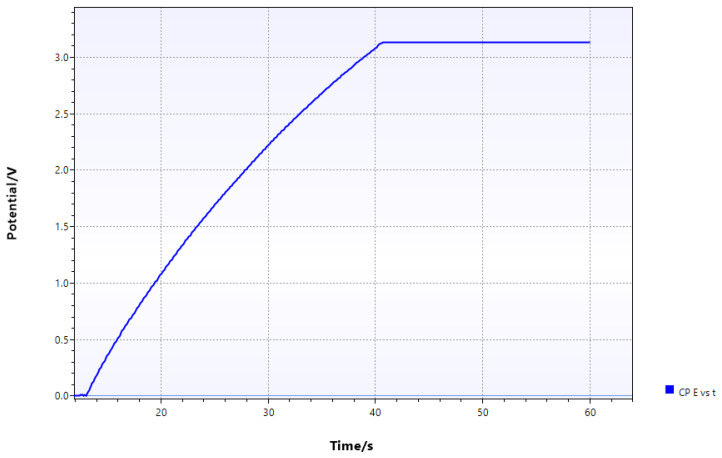
Voltage vs. time plot showing capacitor charging behavior with a 1000 µF capacitor charged to 3.1 V.

A 1000 µF capacitor was charged using the rectified output from the piezoelectric harvester at an optimal frequency of 65 Hz to evaluate power storage capability. The voltage increased smoothly over time until the capacitor reached approximately 3.1 V, as shown in Figure 7.

The energy stored in the capacitor is calculated as follows:

$$E=\frac{1}{2}CV^{2}=\frac{1}{2}\times 1\times 10^{-3}\times {\left(3.1\right)}^{2}=4.80\;\mathrm{mJ}$$

The charging process took about 30 seconds, giving an average power output of

$$P=\frac{E}{t}=\frac{4.80\times 10^{-3}}{30}=160\;\mu W$$

#### Theoretical Power Output and Power Density

The maximum theoretical power output based on the DuraAct transducer’s specified 200 V maximum $V_{rms}$ is estimated as follows:

$$P_{max}=29\;\mu W\times {\left(\frac{200}{1.4}\right)}^{2}\approx 591.84\;\mathrm{mW}$$

Using the transducer dimensions (61 mm × 35 mm × 0.4 mm), the volume is 8.54 ${\mathrm{cm}}^{3}$. Thus, the theoretical power density is

$$\mathrm{Power}\;\mathrm{Density}\;\left(\mathrm{Theoretical}\right)\approx 692.97\;\mu {W/\mathrm{cm}}^{3}$$

For the tested power output of 160 µW, the measured power density is

$$\mathrm{Power}\;\mathrm{Density}\;\left(\mathrm{Tested}\right)\approx 187.35\;\mu {W/\mathrm{cm}}^{3}$$

Theoretical Power Density: 692.97 µW/${\mathrm{cm}}^{3}$; Tested Power Density: 187.35 µW/${\mathrm{cm}}^{3}$

This comparison between theoretical and tested power densities highlights the transducer’s practical performance relative to its maximum potential.

### 3.5. Practical Application: Cyclic Voltammetry Using a Low-Power Potentiostat Powered by the Harvester

#### 3.5.1. Overview of the Low-Power Potentiostat AFE Design

The low-power potentiostat AFE design consists of a custom-designed printed circuit board (PCB) integrated with four LMP91000 AFE modules. These modules are optimized for low-power applications, making them ideal for portable electrochemical sensing tasks such as cyclic voltammetry.

**Figure 8:**
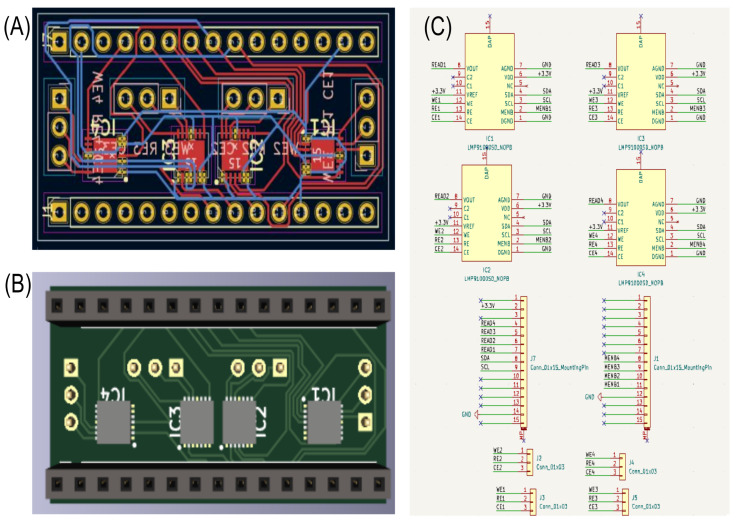
PCB design for the low-power potentiostat AFE. (**A**) Final KiCad design of the PCB layout; (**B**) 3D model of the expected PCB appearance; (**C**) pin layout of the board, showing how the LMP91000 modules are connected. This design highlights the arrangement necessary for compact, low-power operation that can simultaneously support multiple electrochemical sensors.

The design incorporates the following key features:

- Four LMP91000 AFE Modules: The PCB hosts four LMP91000 analog front ends, each handling one sensor for electrochemical measurements. These modules are designed specifically for low-power electrochemical sensing, which fits the needs of portable applications.
- Pin Structure: The pinout is structured to provide flexible connectivity. I2C communication is used to integrate the modules with the Arduino Nano for real-time data acquisition. The layout ensures that each sensor connects smoothly to the necessary pins (WE, RE, and CE) for electrochemical measurement.
- Multi-Communication Capability: The PCB includes an Arduino Nano, which is mounted directly on top of the AFE modules, as seen in the figures. The Arduino provides multiple data transfer options, including Wi-Fi, Bluetooth, and wired connections. These options are ranked in terms of power consumption, with Wi-Fi being ranked the highest, Bluetooth as a medium-power option, and wired communication requiring the least power. This flexibility allows the setup to be tailored for different energy conditions based on the power available from the energy harvester.
- Compact PCB Layout: The PCB layout is optimized for compactness, which helps in maintaining the integrity of signals while ensuring low power consumption. Figure 8 demonstrates the compact and efficient design of the PCB, with the components arranged for minimal space utilization.
- Validation Using a Diode: A diode is integrated into the circuit to verify the accuracy of the cyclic voltammetry sweeps. This validation ensures the precision of the electrochemical measurements, especially when powered by the harvested energy.

In Figure 8, the comprehensive layout for the low-power potentiostat AFE is presented. Subplot (A) shows the final design created in KiCad, providing a detailed view of the PCB’s routing and component placement. Subplot (B) illustrates the expected 3D model of the PCB, offering a visual representation of the finished board. Lastly, subplot (C) details the pin layout, demonstrating the connectivity of the four LMP91000 modules essential for communication and power management. This arrangement underscores the compact and efficient design, which is optimized to support multiple electrochemical sensors while maintaining low power consumption.

Figure 4B shows the integration of the Arduino Nano with the PCB, enabling versatile communication options. Each subfigure contains a scale bar in the bottom-right corner, aiding in the assessment of component sizes and spatial arrangement. This design facilitates flexible data transmission via Wi-Fi, Bluetooth, or cable, making it well-suited for energy harvesting applications.

#### 3.5.2. Capacitor Charging and Powering the Potentiostat System

In this phase of testing, we highlight the use of the energy stored in a capacitor to power the low-power potentiostat system. While in earlier modules, we demonstrated the capability of the energy harvester to charge a capacitor using harvested energy from ambient sources, here, we focus on utilizing the stored energy in the capacitor to operate the potentiostat. This is a crucial step, as it showcases the viability of powering an electrochemical sensing device entirely through energy harvesting.

- Capacitor Charging: The energy harvester module was previously tested and proven effective in charging a capacitor using vibrational energy. This energy is stored in the capacitor, which can later be discharged to power low-power systems. For this experiment, a capacitor with a capacity of 1000 µF was charged to a voltage sufficient to run the potentiostat system.
- Powering the Potentiostat System: Once the capacitor was charged, it was disconnected from the energy harvester and connected to the potentiostat system. The energy stored in the capacitor was then used to power the entire system, enabling it to perform cyclic voltammetry sweeps. The device successfully ran on the stored energy, demonstrating that the system can operate independently of the energy harvester once the capacitor is sufficiently charged.
- Power Considerations: The energy stored in the capacitor was sufficient to power the potentiostat for a defined period, depending on the communication mode used. Wi-Fi, Bluetooth, and wired communication modes were tested, with each mode requiring different amounts of power. Wi-Fi, being the most power-intensive, reduced the operational time compared to Bluetooth or wired connections. This demonstrates that, based on available energy, the system can be optimized to use more efficient communication modes.

By separating the testing of the energy harvester and the capacitor-powered potentiostat system, we were able to validate both the energy harvesting capabilities and the feasibility of operating a real-world application. This method of powering the potentiostat with stored energy opens up new possibilities for autonomous, low-power sensing devices that can operate in remote or energy-constrained environments.

#### 3.5.3. Cyclic Voltammetry Sweep Process

The cyclic voltammetry (CV) sweep process is a key component of the potentiostat’s functionality. Using the low-power design of the LMP91000 AFE modules, this system is capable of performing electrochemical sensing by applying a sweeping voltage across a working electrode (WE) and a counter electrode (CE) and measuring the current response.

The LMP91000 is specifically designed for low-power potentiostat applications and supports three-electrode electrochemical cells. It features a programmable bias voltage, an internal zero, and reference voltage buffers, making it ideal for CV and other electrochemical analysis methods. The key specifications of the LMP91000 include the following:

- A programmable transimpedance amplifier (TIA) with multiple gain settings, allowing for flexibility in detecting a wide range of currents from the electrochemical reactions.
- A 3.3 V supply voltage ($V_{IN}$) and reference voltage ($V_{REF}$), chosen for this application to ensure compatibility with low-power and energy harvesting systems.
- Integrated support for a wide range of sensor types, including amperometric, voltammetric, and potentiometric sensors, enhancing its versatility for cyclic voltammetry measurements.

For the CV sweep, the following key parameters were set:

- Supply and Reference Voltages: Supply ($V_{IN}$) and reference ($V_{REF}$) voltages of 3.3 V were selected. This value ensures that the system remains within the low-power constraints of the energy harvester while providing a sufficient range for the CV sweep.
- Voltage Sweep Range: A cyclic voltammetry sweep was conducted over a potential difference of −24% to +24% of the reference voltage ($V_{REF}$). This translates to a sweep range of approximately −0.792 V to +0.792 V between the working electrode (WE) and the counter electrode (CE). This range was chosen to capture the relevant electrochemical activity in the system while staying within safe operating limits of the energy harvesting setup.
- Electrode Configuration: The system was configured in a standard three-electrode setup, with the working electrode (WE), reference electrode (RE), and counter electrode (CE) connected to the LMP91000 modules. The working electrode experiences the potential sweep, while the current response is measured to evaluate the electrochemical behavior of the analyte.

During the CV sweep, the potentiostat modulated the potential between the working and counter electrodes while measuring the resulting current. This current provides critical information about the redox reactions occurring at the electrode surface, allowing for the detection and analysis of various electrochemical phenomena.

Powering the Process: The energy required to perform the CV sweep was drawn from the charged capacitor, which had been powered by the energy harvester in previous tests. The LMP91000’s low power requirements (operating at 3.3 V) made it possible for the system to run the entire cyclic voltammetry process using stored energy, without any external power sources. The system performed accurately, validating the energy harvester’s ability to sustain such processes.

This experiment demonstrates the successful integration of a low-power electrochemical sensing system with energy harvesting, paving the way for autonomous sensing devices that can operate in energy-constrained environments.

#### 3.5.4. Power Consumption Summary

To assess the feasibility of powering the potentiostat system in different communication modes, we analyzed power consumption and operational cycles. Table 1 summarizes current and power consumption, cycles supported per charge, and daily cycles based on available harvested energy. This comparison highlights that the system performs best in wired mode, while Wi-Fi mode has the highest power demands.

The data in Table 1 reveal that while the LMP91000 operates with minimal power, the Arduino Nano’s consumption increases significantly in Bluetooth and Wi-Fi modes. This analysis suggests that wired communication is the most efficient, supporting the highest cycle count, while Wi-Fi mode is best suited for short-duration operations due to its high power demands.

#### 3.5.5. Validation of Cyclic Voltammetry Accuracy Using a Diode

To validate the accuracy of the cyclic voltammetry sweeps, a diode was used as a simple device under test to ensure system functionality. Diode-based cyclic voltammetry was performed on each of the four potentiostat devices, and the resulting plots are shown below. These plots confirm the correct operation of each potentiostat by demonstrating the expected characteristic curves across the sweep.

A cyclic voltammetry sweep was conducted with a chosen $V_{in}$ of 3.3 V, with $V_{ref}$ set at 3.3 V as well. The sweep was performed between $-24\%V_{ref}$ and $+24\%V_{ref}$ as the potential difference between the working electrode (WE) and the counter electrode (CE).

**Figure 9:**
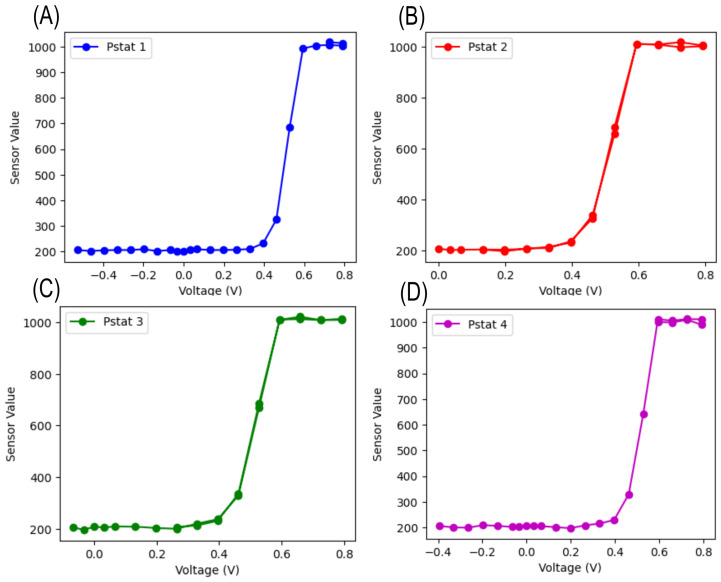
Cyclic voltammetry results for (**A**) Potentiostat 1, (**B**) Potentiostat 2, (**C**) Potentiostat 3, and (**D**) Potentiostat 4. Each curve demonstrates the expected diode response, confirming correct potentiostat functionality.

The results for each potentiostat (Pstat 1 to Pstat 4) are combined in Figure 9, where the sensor value is plotted against the applied voltage. These characteristic curves indicate a clear response around the expected voltage thresholds, confirming that the low-power potentiostat design functions as intended. The successful operation with consistent outputs validates the performance of the system.

Furthermore, the capabilities of the system for wired, Bluetooth, and Wi-Fi communication modes were tested. The system was able to transmit data successfully across all three modes. The corresponding code for handling data transmission in these modes is provided in the Data availability section.

**Figure 10:**
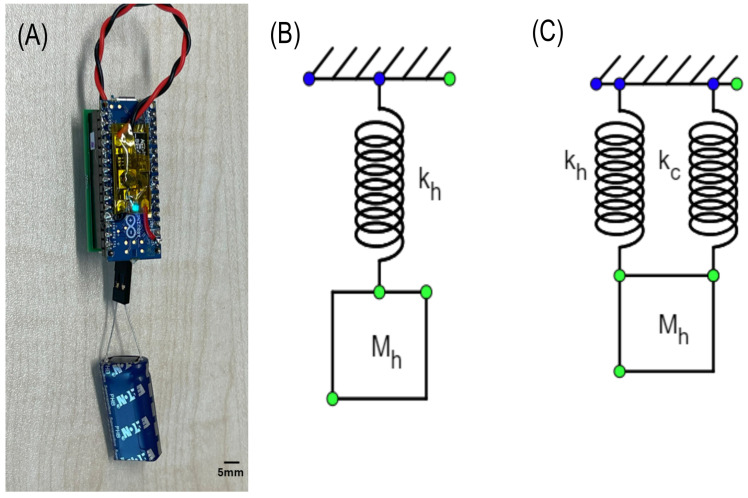
(**A**) Standalone potentiostat system powered by a capacitor charged by the energy harvester. A zoomed-out view shows the complete setup, including the capacitor as the power source. (**B**) Spring-mass equivalent of the harvester as modeled in COMSOL. (**C**) Spring-mass equivalent of the harvester with additional stiffness from the electrical circuit.

Additionally, the standalone functionality of the potentiostat system was validated, as shown in Figure 10. The sensor is powered independently by the capacitor and operates without an external power source. The image highlights the energy harvester’s ability to charge the capacitor and allow the potentiostat to perform its cyclic voltammetry sweeps.

Figure 10 illustrates the standalone potentiostat system, which operates using energy harvested and stored in a capacitor. Subplot (A) gives a broader view of the entire setup, including the capacitor, which functions as the primary power source.

## 4. Discussion

### 4.1. Spring-Mass Equivalent System

To explain the difference between the COMSOL simulation eigenfrequency (35 Hz) and the experimentally measured natural frequency (65 Hz), we need to model the system using a spring-mass-equivalent approach. The system can be represented by two configurations: one corresponding to the harvester modeled in COMSOL without additional circuit components and another that includes the stiffness added by the electrical circuit and connectors.

In Figure 10B, the mass-spring system is simplified, with stiffness ($k_{h}$) corresponding to the harvester and cantilever beam’s stiffness and $M_{h}$ representing the mass of the system (approximately 25 g including the beam, harvester, and magnets). The equivalent natural frequency (*f*) can be calculated using the well-known relation for a spring-mass system:

$$f=\frac{1}{2\pi }\sqrt{\frac{k}{M}}$$

Substituting the given eigenfrequency of f=35Hz and the total mass of M=25g=0.025kg, we can solve for $k_{h}$ (the stiffness of the harvester):

$$k_{h}={\left(2\pi f\right)}^{2}M={\left(2\pi \cdot 35\right)}^{2}\cdot 0.025=12,084\;N/m$$

Now, when the additional electrical circuit is introduced, the system experiences additional stiffness due to the mechanical influence of the wires and connectors. This added stiffness is represented as $k_{c}$ in Figure 10C, and the total stiffness of the system becomes $k_{h}+k_{c}$. The natural frequency of the system increases as a result, and the measured frequency of the prototype is found to be 65 Hz.

Using the new frequency of f=65Hz with the same mass, we can calculate the total stiffness:

$$k_{\mathrm{total}}={\left(2\pi \cdot 65\right)}^{2}\cdot 0.025=26,447\;N/m$$

Given that the total stiffness is the sum of the harvester stiffness and the circuit stiffness,

$$k_{\mathrm{total}}=k_{h}+k_{c}.$$

Substituting the previously calculated value of $k_{h}$,

$$26,447=12,084+k_{c},$$

and solving for $k_{c}$ yields

$$k_{c}=26,447-12,084=14,363\;N/m.$$

Thus, the additional stiffness ($k_{c}$) introduced by the electrical circuit and measurement setup is approximately 14,363 N/m. This explains the observed shift in frequency from 35 Hz (COMSOL model) to 65 Hz (measured frequency in the prototype).

### 4.2. Analysis of the Results

The experimental and simulation results provide several insights into the performance of the piezoelectric harvester. The shape optimization applied to the cantilever beam played a significant role in enhancing energy harvesting efficiency. By fine tuning the beam geometry, the natural frequency of the system was aligned closer to the target operating frequency, enabling more efficient energy conversion from mechanical vibrations. Furthermore, the addition of magnets increased the stress on the beam, leading to greater deformation, which, in turn, resulted in a higher open-circuit voltage. This increase in voltage translated into a greater power output, as more energy could be captured from the mechanical excitation.

The observed maximum power output of 29.1 µW occurred at 65 Hz, as opposed to the 35 Hz predicted by the COMSOL model. This deviation is primarily due to the extra layers in the commercial piezoelectric transducer that were not considered in the simulation. These layers, including insulation pads and protective coatings, added stiffness to the system, causing the actual natural frequency to shift to a higher value. This aligns with the analysis of the spring-mass-equivalent system, where additional stiffness was found to lead to a higher resonant frequency. The electrical measurement setup, including wires and connectors, also contributed to this stiffness, further increasing the system’s effective stiffness and shifting the resonance to 65 Hz.

Moreover, the increased stress due to the magnets resulted in larger displacements at the beam’s tips, which improved energy conversion efficiency. The stress concentration near the magnets, as indicated by the COMSOL model, contributed to a higher-voltage output, as larger strains in piezoelectric materials typically lead to the generation of greater electrical potential.

The harvester’s consistent ability to produce peak power at 65 Hz suggests that the system can be effectively tuned to target specific vibrational frequencies found in real-world environments. This has important implications for applications in automotive engine vibration and low-frequency human motion, where the vibration source may not naturally match the transducer’s frequency. Shape optimization and mechanical adjustments, such as the use of magnets, allow the system to be tuned to the desired operating conditions.

Although the COMSOL model offered valuable insights into the stress distribution and natural frequency, it did not account for real-world complexities present in the physical prototype, such as additional stiffness introduced by external components. Despite these differences, the experimental results demonstrate that the harvester achieves performance to theoretical predictions, particularly regarding its ability to harvest energy at its resonant frequency and efficiently convert it into usable electrical power.

### 4.3. Comparison with Previous Studies

This section compares the results of our study with those from other key studies in the field of piezoelectric energy harvesting. As shown in Table 2, our system demonstrates significantly better performance in terms of power output and power density, even when compared to studies using similar or more intense stimulation conditions.

For example, Lee et al. [19] used hexagonal boron nitride (h-BN) nanoflakes and achieved an open-circuit voltage of 9 V with a power output of 0.3 μW under mechanical bending. While their voltage is higher, their power output is far lower than the 160 μW produced by our system. This demonstrates the improved energy harvesting efficiency achieved through shape optimization and the addition of magnets in our design.

Zhu et al. [20] utilized PLLA nanofibers, reporting an open-circuit voltage of 0.55 V and a power output of 19.5 nW under rotational stimulation at 1800 RPM. In contrast, our system vastly outperforms theirs, with a much higher power output and voltage under vibrational stimulation. This result highlights the advantage of using commercially available DuraAct transducers in combination with mechanical adjustments to optimize energy harvesting.

Siddiqui et al. [21] achieved an open-circuit voltage of 9.8 V and a power density of 13.5 μW/${\mathrm{cm}}^{2}$ with a P(VDF-TrFE)/BT composite under cyclic bending. While their voltage is higher, our system achieves a significantly higher power density of 187.35 μW/${\mathrm{cm}}^{3}$ under 2 g acceleration, underscoring the efficiency of our design in harvesting energy from mechanical vibrations.

Similarly, Yaqoob et al. [22] used a PVDF-BT/n-graphene composite under 2 g acceleration, obtaining a peak open-circuit voltage of 10 V and a power output of 5.8 μW. In comparison, our system generated 160 μW of power under the same acceleration, highlighting the superior performance of our optimized cantilever beam with magnets.

Alam and Mandal [23] reported an open-circuit voltage of approximately 30 V and a power density of 9.0 μW/${\mathrm{cm}}^{3}$ using a PDMS/MWCNT composite under repeated hand punching. Although their voltage is higher, our system’s power density is much greater, demonstrating its higher energy conversion efficiency, even under lower mechanical stress conditions.

Bhavanasi et al. [24] used a P(VDF-TrFE)/GO composite, reporting a voltage of 4 V and a power density of 4.41 μW/${\mathrm{cm}}^{2}$. Again, our system far exceeds this, with a power density of 187.35 μW/${\mathrm{cm}}^{3}$, confirming the benefits of shape optimization and material selection in enhancing energy harvesting efficiency.

Ye et al. [25] reported a peak output voltage of 22 V and a power density of 11.3 μW/${\mathrm{cm}}^{2}$ using a P(VDF-TrFE)/BNNT composite under 0.4 MPa pressure. Despite their higher voltage, our system achieves a significantly higher power density, confirming the superior energy harvesting capability of our design under vibrational conditions.

Our study demonstrates a clear advantage in both power output and power density over many existing systems. In addition to superior performance, a key strength of our system lies in its portability and versatility. The compact and lightweight design makes it suitable for integration into portable and wearable devices. Furthermore, the modularity of the system allows for easy customization to target different energy sources. By adjusting the cantilever’s shape or modifying the magnets, our energy harvester can be tailored to harvest energy from a range of vibrational environments, making it highly adaptable for different applications.

The adaptability and efficiency of our system are significant advantages over many of the systems listed in Table 2. While those systems may be optimized for specific applications or materials, our design’s flexibility allows it to be applied across a wider range of environments. For instance, Alam and Mandal’s device relies on repeated hand-punching mechanisms, limiting its applicability, while our system can operate under various vibrational conditions without requiring significant changes to the design.

Our work outperforms many other systems in terms of power output, power density, and overall versatility. The combination of shape optimization, material selection, and mechanical enhancements such as the addition of magnets enabled the creation of a highly efficient, portable, and adaptable energy harvesting system. This makes it an ideal solution for powering low-power electronics and portable devices across a wide range of environments.

### 4.4. Novelty of the Study

#### 4.4.1. Structural Optimization with Commercial Components

Unlike many studies that rely on custom-built designs, this work optimizes a commercially available DuraAct piezoelectric transducer by adjusting the cantilever beam geometry. This approach enables frequency tuning to target specific vibrational sources without complex structural alterations.

#### 4.4.2. Integrated Use of COMSOL and Equivalent Spring-Mass Models

By combining COMSOL simulations with an equivalent spring-mass model, this study effectively aligns the natural frequency of the harvester with the target frequency. This integration allows for precise analysis of stress distribution, displacement, and natural frequency, which are critical for maximizing energy output.

#### 4.4.3. Adaptable Design for Varied Vibration Sources

The adaptable cantilever structure allows for tuning across different vibrational environments. For instance, by adding tip mass (magnets) to the cantilever, the system’s resonant frequency is adjusted to align with lower-frequency sources, such as household and automotive vibrations.

#### 4.4.4. Simplified, Cost-Effective Development Path

The use of readily available materials and components provides a practical framework for researchers and practitioners to develop application-specific harvesters without the need for expensive modifications. This makes the design not only efficient but also economically viable.

#### 4.4.5. Enhanced Performance Metrics

The optimized system achieves high power density and power output metrics, with tested power density reaching 187.35 μW/${\mathrm{cm}}^{3}$ under 2 g acceleration. These metrics demonstrate a significant improvement over comparable systems, validating the effectiveness of the shape and mass modifications in enhancing energy conversion efficiency.

This study’s methodology offers an accessible, adaptable framework for optimizing piezoelectric energy harvesters, making it highly applicable for real-world applications such as automotive sensors, household devices, and wearable electronics.

### 4.5. Implications of the Findings

The broader implications of our findings highlight the practicality and adaptability of piezoelectric energy harvesting systems, especially when leveraging commercially available transducers such as the DuraAct patch used in our study. One of the key advantages of our approach is the simplicity and efficiency of integrating shape optimization techniques with widely accessible components. This combination allows for rapid, cost-effective development of energy harvesters tailored for specific applications without the need for complex, custom-built systems.

Our system operates at 65 Hz, which aligns closely with the frequency of common household devices such as clothes dryers and vacuum cleaners. The operational frequency of our prototype makes it particularly well-suited for energy harvesting in environments where these appliances are used, tapping into their vibrations as a reliable energy source. Moreover, with a high power density of 187.35 μW/${\mathrm{cm}}^{3}$ and the potential to achieve even greater power outputs, this system outperforms many other energy harvesting solutions currently available.

The versatility of the proposed design approach allows for the use commercially available transducers, which reduces the development cost and time to deploy portable energy harvesting devices and systems that can be applied across a broad range of industries for real-world applications. For example, our system could easily be adapted for wearable electronics technologies by designing its frequency range to capture the lower-frequency vibrations generated by human activities, as shown in Figure 1. Similarly, the system can target higher frequencies to harvest energy from industrial equipment, vehicle engines, or even marine environments. This adaptability further underscores the potential for widespread adoption of our method in different sectors.

Additionally, the high energy density of our prototype ensures that its application devices can operate for extended periods without relying on external power sources.

The combination of simplicity, high performance, and ease of adaptation to different frequency ranges makes our approach an appealing option for industries looking to adopt piezoelectric energy harvesting technology. Whether targeting human activity, industrial machinery, or household appliances, our system’s versatility offers a cost-effective and efficient solution for a wide array of energy harvesting applications.

#### 4.5.1. Potential Applications and Unique Advantages

The low-power potentiostat setup, powered by an energy harvester and a capacitor, offers several real-world applications, particularly in scenarios where energy efficiency, portability, and sustainability are critical. By utilizing an energy harvester to charge a capacitor, then power the potentiostat system, we can leverage multiple sources of mechanical energy available in the ambient environment, in combination with other energy harvesting technologies for different sources such as solar energy [26] or body heat [27], to create self-sustaining devices. This makes the system ideal for remote and off-grid applications.

##### Wearable and Portable Health Monitoring Devices

One of the most promising applications for this system is in the field of wearable health monitoring devices. These devices can utilize the energy harvested from human motion or environmental sources to power electrochemical sensing for real-time health data acquisition. For example, the potentiostat could be integrated into a wearable device for continuous glucose monitoring, lactate sensing, or detection of other biomarkers. By reducing the need for frequent charging and battery replacement, this setup enhances user convenience and device longevity, all while maintaining accurate sensing capabilities.

##### Environmental Monitoring

The potentiostat setup can also be adapted for environmental monitoring applications, such as detecting pollutants or heavy metals in water sources. In remote locations where access to electrical infrastructure is limited, the energy harvester provides a sustainable solution for continuously operating sensors to monitor water quality. The ability to communicate data via Wi-Fi or Bluetooth allows for real-time reporting and analysis without the need for constant human intervention.

##### Industrial and Agricultural Sensing

In industrial settings, the potentiostat system can be used for corrosion monitoring, chemical process control, or gas sensing. These applications typically require continuous data collection in environments where power supply interruptions can be costly. Similarly, in agriculture, the system can be used to monitor soil quality or detect nutrient levels, with the added advantage of energy autonomy.

##### Advantages of the Energy Harvester-Powered Setup

- Energy Autonomy: The key advantage of powering the potentiostat with an energy harvester is its ability to operate independently of conventional power sources. This autonomy allows the system to function in remote or inaccessible locations, providing a reliable and sustainable solution for long-term monitoring.
- Extended Operation Time: By integrating a capacitor with high capacitance, such as 250 F at 3.8 V, the system can store a significant amount of energy. This allows the device to run multiple cyclic voltammetry sweeps and communicate data through wired, Bluetooth, or Wi-Fi modes. The modular nature of the setup enables it to adjust power consumption based on the selected communication mode, optimizing energy usage and extending operation time.
- Scalability and Flexibility: The system is highly flexible and can be scaled up for more complex sensing tasks or adapted for various energy harvesting methods. Whether deployed in a small wearable device or a larger industrial monitoring system, the potentiostat setup can be modified to meet specific needs without compromising on power efficiency or sensor accuracy.
- Low Power Consumption: The low-power design of the LMP91000 potentiostat AFE and the careful selection of communication modes (cable, Bluetooth, or Wi-Fi) enable the system to minimize energy consumption while maintaining accurate sensing performance. This makes the setup suitable for energy-sensitive environments.
- Sustainability and Reduced Maintenance: By eliminating the need for batteries or external power sources, this energy harvester-powered system significantly reduces maintenance requirements and environmental impact. In long-term monitoring applications, this leads to lower operational costs and a more sustainable solution.

In summary, the energy harvester-powered potentiostat setup demonstrates its versatility and efficiency across a wide range of applications, from healthcare and environmental monitoring to industrial sensing. Its autonomy, low power consumption, and adaptability make it a highly advantageous solution for modern sensor networks and portable monitoring systems.

### 4.6. Limitations of the Study

Although this study demonstrates the significant potential of piezoelectric energy harvesting using commercially available components, there are a few limitations to acknowledge. One notable limitation arises from the electrical measurement setup. Each measurement setup introduces different levels of stiffness and mass to the system, which can alter the natural frequency slightly. This additional stiffness caused a shift in the prototype’s operating frequency compared to the COMSOL model. However, this limitation can be compensated for by performing additional testing to recalibrate the system and ensure accurate measurements, thereby mitigating the impact of the measurement setup on overall system performance.

In terms of simulation accuracy, because the real-world prototype includes added layers, such as insulation or bonding materials, the additional stiffness they introduce was not captured in the simulation. As a result, while the COMSOL model predicted the system’s natural frequency accurately in an idealized scenario, the prototype’s frequency was shifted due to these unmodeled elements. This difference can be addressed in future studies by incorporating a more comprehensive spring-mass model that accounts for the extra stiffness introduced by the setup, ensuring closer alignment between the simulation and actual performance.

Despite these minor limitations, the advantages of this study far outweigh the disadvantages. The use of commercially available components simplifies the process of producing energy harvesters for specific applications, making the approach more accessible, cost-effective, and easy to replicate. Additionally, the high power density and overall power output of the system, as demonstrated in comparison to other studies, highlight its practical potential for real-world applications. The prototype has proven to be versatile and capable of operating in a wide range of environmental conditions, from low-frequency human activities to high-frequency industrial vibrations.

## 5. Conclusions and Future Work

### 5.1. Conclusions

In this study, we successfully demonstrated the potential of using commercially available piezoelectric transducers to create an efficient and adaptable energy harvesting system. By employing simple yet effective techniques such as shape optimization and the addition of magnets, we were able to achieve notable power outputs and high power densities, outperforming several systems detailed in previous studies. The ability to achieve a maximum power output of 160 µW and a power density of 187.35 µW/${\mathrm{cm}}^{3}$ while operating at a natural frequency of 65 Hz emphasizes the effectiveness of our approach. Furthermore, with the theoretical maximum power density reaching 692.97 W/$m^{3}$, this system has the potential to generate even higher outputs under optimal conditions.

One of the standout features of this research is the versatility and portability of the energy harvesting system. The use of commercially available transducers makes it possible to rapidly produce systems for various specific applications, reducing both time and cost. This system can be easily adapted to target different energy sources and environments, from human activities to industrial vibrations, as demonstrated by the range of potential applications depicted in Figure 1. In particular, our prototype has shown strong compatibility with vibration sources such as clothes dryers, which operate at frequencies around 60 Hz, making it highly suitable for consumer and industrial applications alike.

Despite some limitations, such as the influence of the measurement setup on system stiffness and the challenges posed by discrepancies between the COMSOL model and the real prototype, we believe that the advantages of this system outweigh its limitations. This study revealed that the additional stiffness introduced by the electrical setup can be compensated for through testing and adjustment, while the COMSOL simulations provided valuable insight into stress distribution and resonance behavior.

Looking ahead, there is significant potential for further optimization and exploration in this field. Future research could focus on custom-built, multi-layered transducers that are designed specifically to maximize power output in targeted applications. The integration of nonlinear energy harvesting mechanisms and hybrid systems combining piezoelectric, solar, and thermal energy could lead to even more efficient and self-sustaining solutions. Furthermore, testing the long-term performance and reliability of this system in real-world environments, such as automotive or wearable applications, will provide additional validation of its practicality and effectiveness.

This work contributes to the growing body of research aimed at developing low-power, self-sustaining systems that can operate autonomously in a variety of environments. The flexibility, efficiency, and ease of production of our piezoelectric energy harvester make it an attractive solution for future energy needs, particularly in the context of the Internet of Things and other emerging technologies. The high power density, combined with the adaptability of the system, positions this technology as a key player in the ongoing transition toward more sustainable and efficient energy sources.

### 5.2. Future Research Directions

The results of this study present a strong foundation for future research in piezoelectric energy harvesting. One potential avenue for future work is the optimization of transducer design. While this study utilized commercially available components, future research could focus on custom-built, multi-layered structures tailored for specific applications. These custom structures could integrate multiple piezoelectric layers or hybrid materials, which have the potential to significantly increase power output and energy conversion efficiency. By experimenting with different material compositions and geometries, researchers could optimize both the mechanical and electrical properties of the transducer to maximize its energy harvesting capabilities.

Additionally, more advanced energy harvesting mechanisms, such as nonlinear vibration absorbers, could be explored to enhance the system’s adaptability across a wider range of frequencies. Nonlinear systems are known for their ability to harvest energy over a broad frequency spectrum, making them ideal for real-world applications where vibration sources may not be consistent or predictable. Incorporating such mechanisms could allow energy harvesters to be more resilient in environments with fluctuating or multi-modal vibrational energy, improving overall energy capture and performance.

Yet another promising direction is to investigate how environmental factors, such as temperature, humidity, and exposure to different types of vibrations, impact the system’s performance. This is particularly relevant in scenarios where the energy harvester may be deployed in harsh or variable conditions, such as in marine environments or industrial settings. Understanding the environmental limitations of the system will help ensure its durability and reliability in long-term use.

Further testing of the system in real-world applications, such as automotive environments or wearable devices, is also crucial. In an automotive context, for example, the energy harvester could be used to capture vibrations from vehicle engines, wheel movements, or road surfaces, providing a sustainable power source for low-power electronic sensors. Similarly, in wearable devices, the harvester could convert human body movements into electrical energy, powering sensors or communication devices without the need for batteries.

Another important research direction would be to explore scalability and integration in larger systems. As energy harvesting technologies advance, there is potential to scale up the system for larger applications, such as powering distributed sensor networks in smart cities or augmenting renewable energy systems. In parallel, integrating the harvester with energy storage systems, such as capacitors or supercapacitors, could ensure a more consistent energy supply, allowing the system to store energy during periods of low vibration and discharge it when needed.

Finally, advancements in modeling and simulation techniques, such as the coupling of finite element analysis with real-time experimental data, could refine our understanding of the system’s dynamics and provide more accurate predictions of its performance across different applications. Future studies could benefit from incorporating more sophisticated modeling techniques that account for factors like additional layers, setup stiffness, or variable material properties, ensuring that the theoretical models align more closely with real-world performance.

In summary, the future of piezoelectric energy harvesting research holds great potential. By optimizing transducer design, exploring nonlinear energy harvesting mechanisms, understanding the effects of environmental conditions, and conducting further testing in diverse applications, researchers can continue to improve the efficiency, reliability, and applicability of this promising technology.

## Figures and Tables

**Table 1:** Power consumption, cycles per charge, and daily cycles for different communication modes.

Component / Mode	Current Consumption	Power Consumption	Cycles Per Charge	Daily Cycles
LMP91000	(10 μA) at 3.3 V	(33 μW)	29 cycles	87 cycles
Arduino Nano—Wired	(19 mA) at 3.3 V	(62.7 mW)	15 cycles	44 cycles
Arduino Nano—Bluetooth	(40 mA) at 3.3 V	(132.0 mW)	7 cycles	21 cycles
Arduino Nano—Wi-Fi	(70 mA) at 3.3 V	(231.0 mW)	4 cycles	11 cycles

**Table 2:** Quantitative Comparison of key studies in piezoelectric energy harvesting.

Study	Material	Stimulation	Open-Circuit Voltage (V)	Power Density
Lee et al. [19]	Hexagonal boron nitride (h-BN)	0.05 m/s	9.0	0.033 μW/${\mathrm{cm}}^{2}$
Zhu et al. [20]	PLLA nanofibers	1800 rpm	0.55	Roughly 19.5 nW/${\mathrm{cm}}^{2}$
Siddiqui et al. [21]	P(VDF-TrFE)/BT	x	9.8	13.5 μW/${\mathrm{cm}}^{2}$
Yaqoob et al. [22]	PVDF-BT/n-graphene	2 g	10.0	Roughly 5.8 μW/${\mathrm{cm}}^{2}$
Alam and Mandal [23]	PDMS/MWCNT	40 kPa(punching)	∼30	9.0 μW/${\mathrm{cm}}^{3}$
Bhavanasi et al. [24]	P(VDF-TrFE)/GO	4 mm at 1 Hz	4.0	4.41 μW/${\mathrm{cm}}^{2}$
Ye et al. [25]	P(VDF-TrFE)/BNNT	0.4 MPa	22.0	11.3 μW/${\mathrm{cm}}^{2}$
Present Study	DuraAct PZT Transducer	2 g	3.1	160 μW, 187.35 μW/${\mathrm{cm}}^{3}$

## Data Availability

The data supporting this study are available from the Apollo repository and can be accessed from https://doi.org/10.17863/CAM.113126.
